# Circumstances of suicide after registration with a national digital mental health service: an analysis of coroners’ reports

**DOI:** 10.1192/bjo.2023.60

**Published:** 2023-05-24

**Authors:** Olav Nielssen, Lauren Staples, Eyal Karin, Katie Ryan, Rony Kayrouz, Blake Dear, Shane Cross, Nickolai Titov

**Affiliations:** Faculty of Medicine and Health Science, Macquarie University, Sydney, Australia; and MindSpot Clinic, Sydney, Australia; eCentreClinic, Macquarie University, Sydney, Australia; and MindSpot Clinic, Sydney, Australia; Faculty of Medicine and Health Science, Macquarie University, Sydney, Australia; MindSpot Clinic, Sydney, Australia

**Keywords:** Suicide, risk, MindSpot, digital mental health service, coroner

## Abstract

**Background:**

Little is known about the safety of mental healthcare provided remotely by digital mental health services (DMHS), which do not offer face-to-face contact.

**Aims:**

To examine the circumstances of suicide by patients registered with a national DMHS.

**Method:**

Data from 59 033 consenting patients registered with a national DMHS, the MindSpot Clinic, between 1 January 2013 and 31 December 2016 were linked with the Australian National Death Index and documents held by the National Coronial Information System (NCIS). Data extracted included demographic information, the nature of contact, duration between last contact and death, symptom scores and information in police, autopsy, toxicology and coroners’ reports.

**Results:**

Of the 59 033 patients, 90 (0.15%) died by suicide in a follow-up period of up to 5 years. The mean time between last contact and death was 560 days. Coroners’ reports were located for 81/90 patients. Most (87.0%) were receiving face-to-face care around the time of death, 60.9% had a documented previous suicide attempt, 52.2% had been in hospital in the previous 6 months and 22.2% had severe mental illness, mainly schizophrenia or bipolar disorder. Other common findings were current treatment with psychotropic medication (79.2%) and the presence of alcohol (41.6%), benzodiazepines (31.2%), and illegal drugs and non-prescribed opioids (20.8%) at time of death.

**Conclusions:**

Those who died by suicide after contact with the DMHS had more severe illness, were mostly engaged with face-to-face services and often had disinhibiting substances, especially benzodiazepines, present at the time of death.

Digital mental health services (DMHS) were developed to increase access to evidence-based care for people with high-prevalence disorders such as anxiety and depression^1,2^ and they now provide remote treatment as part of routine care in several high-income countries. The effectiveness and efficiency of DMHS are well-established, with clinical outcomes that are comparable to high-quality face-to-face care at a fraction of the cost.^3–5^ Depending on the location and the nature of the DMHS, as many as one-third of patients registering with DMHS report that they had never received any kind of mental healthcare.^4,6^

Australia has several DMHS, including the MindSpot Clinic (MindSpot), funded by the Australian Department of Health and Aged Care, which provides evidence-based assessment and treatment by trained mental health professionals to over 20 000 adults with symptoms of anxiety, depression and chronic pain each year.^4,7,8^ Most clinic patients self-refer, with only a minority referred by clinicians who have seen the patients in person. In contrast, other Australian DMHS either only take referrals from general practitioners and other clinicians^9,10^ or encourage referrers to use the DMHS alongside face-to-face treatment.^11^ It had been assumed that the treatment programmes offered by DMHS would be suited to people with mild to moderate symptoms, but three-quarters of MindSpot's patients report clinically significant symptoms of depression, often in the severe range, more than one-quarter report suicidal thoughts, nearly 4% report a current suicide plan^4^ and around 0.5% are referred for urgent face-to-face care.^12,13^

A large prospective study of people who died by suicide in the USA found that about half had been diagnosed with a mental health condition or received treatment in the previous year.^14^ However, a meta-analysis of studies of contact with mental health services at the time of suicide found that only about a quarter of people who died by suicide were in contact with a mental health service at the time of death,^15^ although that analysis did not include any studies of death by suicide after contact with DMHS. A large study conducted in primary and out-patient care found that patients reporting frequent thoughts of death or self-harm on the Patient Health Questionnaire (PHQ-9) were five times more likely to die by suicide in the following year, compared with patients not reporting those thoughts.^16^ A recent systematic review and meta-analysis of internet-delivered cognitive–behavioural therapy to reduce suicidal ideation included six eligible trials and found a significant reduction in suicidal ideation, but noted that the studies did not report sufficient data to assess the effect on suicide attempts or death by suicide,^17^ and to our knowledge this is the first study to examine the circumstances of suicide after contact with a DMHS.

The automation of aspects of assessment and treatment delivery allows DMHS to assess and treat very large numbers of patients, but comparatively little is known about the safety of DMHS, in particular the number and circumstances of deaths by suicide after contact with DMHS and the adequacy of procedures for identifying and managing risk of patients provided with remote mental health services. A preliminary study that matched MindSpot patients from the first 4 years of operation with the Australian Institute of Health and Welfare's National Death Index found that 64 of 59 033 people (0.11%) who registered with MindSpot had died by suicide within 2 years of last contact, including 7 within 30 days and a further 7 within 90 days of contact.^13^ A review of the files of those patients found that clinic safety protocols were followed in every case, that only 4 of the 285 urgently referred to local mental health or emergency services subsequently died by suicide and only one of those patients died within 90 days of last contact with the clinic. However, the National Death Index did not include information about the circumstances of those who took their life. Furthermore, the clinic records often included only basic demographic information and the results of automated symptom questionnaires for those patients who did not complete their assessments (20%) or who could not be contacted by telephone or email. To establish the circumstances of each case, and whether the interventions to ensure the safety of patients accessing a high-volume partly automated DMHS were adequate, an application was made to access data held by the National Coronial Information System (NCIS).

The purpose of this study was to identify the risk profile of patients using a DMHS to inform the further development of appropriate clinical standards for this model of service delivery. Hence the aims of this study were to (a) identify the circumstances of suicide of patients who had registered with MindSpot and (b) establish the factors associated with death by suicide. This paper is an extension of an earlier study that examined data held by the clinic for those known to have died by suicide.^13^ There is inevitably some overlap in the methods and results, but the present study expands on the previous study in examining the circumstances of death in most cases using data from coronial investigations.

## Method

### Patients and data linkage

**Fig. 1:**
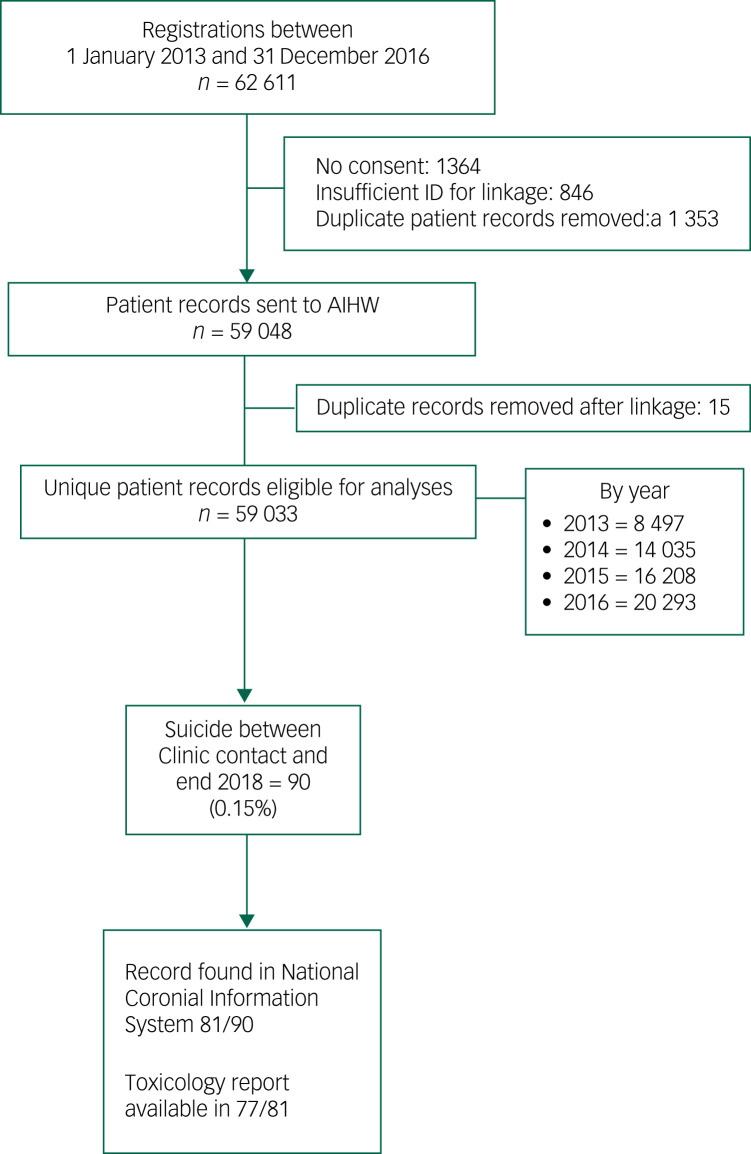
Patient flow diagram. a. Duplicate patient records – a total of 1353 started more than one assessment (range 2–8). In cases where the linkable (identifiable) data were identical for each assessment, only one record was sent to the Australian Institute for Health and Welfare (AIHW). Where variation of identifiable information occurred across assessments (e.g. changes to postcode or variants of first name), these known duplicates (*n* = 16) were included in the records sent to AIHW.

MindSpot provides free assessment and treatment to people identifying as Australian residents aged 18 years or older. A total of 61 611 people registered with MindSpot between 1 January 2013 and 31 December 2016 (Fig. 1), of whom 59 033 provided consent for their records to be used for research purposes and sufficient data for linkage. Following approval from the Human Research and Ethics Committees of Macquarie University (MQ HREC) details of these patients were forwarded to the Australian Institute for Health and Welfare (AIHW) to match with the National Death Index to establish the fact of death and the cause of death using ICD-10 codes, including death by suicide (X60–84). Further ethical approval was obtained to reidentify those patients, and the files of patients who had registered with MindSpot in the first 4 years of operation and who were found to have died by suicide up to the end of 2018 were then examined. In an additional step and after receiving further ethical approval, the files held by the NCIS were searched for information about the circumstances of death.

### Ethics review

Approval to conduct the study was obtained from the MQ HREC (reference no.: 5201949936957), the AIHW (EO2019/2/242), the Justice and Human Research Ethics Committee (CF/21/2961) and the Coroner's Court of Western Australia (EC 20/2021). The MindSpot Clinic is registered on the Australian and New Zealand Clinical Trials Registry (ACTRN12613000407796). The authors assert that all procedures contributing to this work comply with the ethical standards of the relevant national and institutional committees on human experimentation and with the Helsinki Declaration of 1975, as revised in 2008.

### MindSpot Clinic safety protocols

MindSpot operates with a clinical governance framework and policies and procedures aligned with the Australian National Safety and Quality Digital Mental Health Standards.^18^ MindSpot's procedures for identification and management of patients at risk of harm to self or others are based on those of the Department of Health in the state of New South Wales,^19^ adapted for a DMHS. Assessment of risk is based on patient responses to symptom questionnaires and clinical enquiry by trained therapists about thoughts of self-harm and other indicators of risk at assessment and at regular intervals during treatment. Individuals identified as being at risk are asked in more detail about symptoms and the presence of known risk and protective factors, and those who are unable to confirm their safety are referred to local crisis or emergency services. The details of safety procedures and the nature of urgent referrals have been published elsewhere.^12^

### Measures

#### Demographic information

As part of the registration and assessment process, 23 demographic and symptom variables are collected. The demographic information includes age, gender, marital status, country of birth (Australia or other), Indigenous status, employment status and level of education.

#### Symptom questionnaires and clinical indicators of risk

All patients are administered the 9-Item Patient Health Questionnaire (PHQ-9),^20^ the 7-Item Generalized Anxiety Disorder scale (GAD-7)^21^ and the 14-question version of the Kessler Psychological Distress Scale (K10+)^22^ at assessment, and for those who enrol in a MindSpot treatment course, symptom questionnaires are re-administered at weekly intervals during the 8 weeks of treatment, at the conclusion of treatment and again at 3-month follow-up. Patients are also asked series of questions about their past and current health service use, including whether they have ever seen a mental health professional for symptoms of depression or anxiety, whether they speak with a general practitioner about their mental health and whether they are taking psychotropic medication. There are further questions about the reasons for contacting MindSpot and whether the patient is experiencing current difficulties in employment, relationships, physical health or finances. The final questions relate to the presence of suicidal thoughts or whether they have a suicide plan, in addition to question 9 of the PHQ-9.

#### Clinical data extraction

Clinic records were examined to establish the nature of contact (assessment or enrolment in treatment) and the date of last contact. Contact was classified as (a) individuals who commenced an assessment only (*n* = 48 110), (b) individuals who enrolled in a treatment course (*n* = 10 638) and (c) individuals who were urgently referred to an emergency service (*n* = 285) either at the time of assessment (*n* = 252) or during treatment (*n* = 33). Information about the service, the measures used at assessment and during treatment, the nature of the treatment courses offered, patient information and treatment outcomes has been published in detail elsewhere.^4,7,8^ The duration between the date of last contact and death was calculated in days.

#### NCIS data

The NCIS records comprised reports from investigating police, autopsy, toxicology and the coroners’ decisions, including in some cases the reports of full coronial inquests. The coroners’ verdicts were used to establish the fact of suicide, and those who died by self-poisoning with uncertain intent were not included. Searches of the NCIS were conducted by jurisdiction, name, gender, date of birth and date of death. Data extracted included diagnosis, known depressive illness, previous suicide attempt, documented admission to a psychiatric hospital within the last 6 months, contact with mental health services at time of death, including with a general practitioner prescribing psychotropic medication, and recent separation or bereavement. Toxicology reports were also examined for prescribed medication (especially benzodiazepines), alcohol, and illegal drugs and non-prescribed opioids.

### Statistical analyses

The demographic and symptom information was compared with the benchmark reported for the first 7 years of the clinic's operation,^4^ and the frequency of medication use was compared with the medication use reported in a recent large sample of clinic users.^23^

A two-step cluster analysis^24^ was employed to examine the possibility of concurrence among patient characteristics, such as known diagnoses (substance use disorder, depression and/or borderline personality disorder, psychotic illness and/or bipolar disorder), medical history (previous suicide attempt, recent hospital admission), toxicology (any prescribed psychotropic medication, antipsychotic/mood stabiliser, tricyclic antidepressant, benzodiazepine, alcohol present) and two demographic variables (age, gender). Examining cluster patterns of these patient features aimed to determine the possibility of an underlying risk profile. Participant feature concurrence was determined using a log-likelihood distance measure, with the number of clusters automatically determined using the Bayesian information criterion.^25^

Statistical analyses were performed using SPSS version 27 for Windows, and *P* < 0.05 was considered statistically significant for all tests.

## Results

### Number and rates of suicide

A total of 59 033 individuals registered for an online assessment in the first 4 years of operation of the service (Table 1). Linking the registrants with the National Death Index revealed that 90 of those individuals died by suicide between the commencement of operation at the beginning of 2013 and the end of 2018. The mean duration between last contact and death was 560 days (median 490 days, range 3–1803 days). To estimate the rate of suicide we examined suicides within 2 years of last contact with the service.^13^ There were 64 suicides in the 2 years since last contact, equivalent to an age-standardised suicide rate of 56.6 (95% CI 54.0–58.5) per 100 000 per year, which was nearly five times the rate of suicide in Australia in those years.^26^

**Table 1:** Characteristics of patients who died by suicide, irrespective of proximity of contact with MindSpot, compared with a benchmark sample

	Study sample	Benchmark comparison^a^
Number of patients	*n* = 90	−
Number of assessments completed	73/90 (81%)	−
Started a treatment course	10/90 (11%)^b^	
Demographics (at time of assessment)		
Mean age, years	38.2	35.7
Male gender, % (95% CI)	52 (41.7–62.3)	27%
Born in Australia, % (95% CI)	82 (74.1–89.9)	78%
Aboriginal or Torres Strait Islander, % (95% CI)	4.7 (0.3–9.1)	3.7%
Paid employment, % (95% CI)	44 (33.7–54.3)	57%
University degree, % (95% CI)	25 (16.1–33.9)	39%
Married/de facto relationship, % (95% CI)	27 (17.9–36.1)	38%
Symptoms (at assessment)		
K10+ score, mean (s.d.)	36.5 (7.3)	31.8 (7.5)
PHQ-9 score, mean (s.d.)	19.9 (5.6)	14.9 (6.2)
GAD-7 score, mean (s.d.)	15.0 (4.8)	12.5 (5.2)
Score of 3 on item 9 of PHQ-9	35% (27/78)	−
Reported suicidal thoughts, % (95% CI)	66 (56.2–75.8)	32%
Reported suicide plan (of those reporting suicidal thoughts), % (95% CI)	25 (16.1–33.9)	4%
Service use (at assessment), % (95% CI)		
Previous or current health professional	87 (80.1–93.9)	65%
Speaks with a GP about mental health	68 (58.4–77.6)	47%
Current psychotropic medication	48 (37.7–58.3)	27%
Main purpose for seeking service at MindSpot, % (95% CI)		
Assessment and information	50 (39.7–60.3)	67%
Treatment	43 (32.8–53.2)	26%
Other	7 (1.8–12.2)	7%
Main reason for using an online mental health service rather than traditional service, % (95% CI)		
Convenience and cost	43 (31.6–54.4) (31/73)	34%
Privacy and anonymity	17 (8.4–25.6) (15/73)	33%
Other^c^	40 (28.8–51.2) (27/73)	33%

a. The comparison column shows data from a 7-year MindSpot Clinic sample (*n* = 96 018) reported in Titov et al, 2020 (https://doi.org/10.1016/S2589-7500(20)30224-7).^4^

b. Only two patients completed all five lessons of a MindSpot treatment course. Both patients also completed 3-month follow-up. At follow-up, scores on the PHQ-9 and GAD-7 for both these patients were below clinical thresholds.

c. Of the patients who reported other reasons for seeking online support, 26% (7/27) reported that face-to-face services had not helped them or they needed additional care.

A significant number of registrants (11 902 of 59 033, 20.2%) did not fully complete the assessment questionnaires and hence data were incomplete for a large part of the sample. Missing data on any patient variables were included in all analyses as a distinct category in the clinic benchmark sample.^4^

### Characteristics of patients who died by suicide

Of the total sample, 10 671 (18.1%) proceeded to enrol in a treatment course, compared with 10/90 (11%) of those who died by suicide. Those who died by suicide were on average older and less likely to be employed and have a university degree, but were more likely to be married or in a de facto relationship. Not surprisingly, they had higher scores on the K10+, PHQ-9 and GAD-7, and they were also twice as likely to report thoughts of suicide (66% *v*. 32%) and six times as likely to report a suicide plan (25% *v*. 4%). The patients who died by suicide were also more likely to report having seen a mental health professional, report speaking to a general practitioner about mental health and to report taking psychotropic medication (Table 1).

### Data from the NCIS

Documents were found in the NCIS for 81/90 of the patients known to have died by suicide; these included police reports, autopsy reports, toxicology reports, coroners’ decisions and in some cases the reports of coronial inquests. Since the AIHW data on cause of death came from the various state coroners, the missing documents were because the cases had not been closed or had not been uploaded to the NCIS for reasons that included that they contained sensitive information. In a further 12 cases, the documents did not include a detailed psychiatric history or account of the recent circumstances of the deceased, and the only information available was the coroner's summary finding as to the cause of death, with either a brief police report or a brief narrative in the autopsy report. However, 77 of the 81 cases in which documents were available included a toxicology report.

### Clinical information from NCIS documents

The NCIS documents confirmed a high rate of diagnosed depressive disorder, including that associated with borderline personality disorder (81.5%), and a very high rate of severe mental illness, including schizophrenia, bipolar disorder and disabling obsessive–compulsive disorder (22.2%), among those who died by suicide. The reports showed that nearly two-thirds (60.9%) had made a previous suicide attempt and about half (52.2%) had been admitted to hospital in the previous 6 months, including a number who were current in-patients or who had only recently been discharged. The reports also confirmed that a high proportion were currently engaged in face-to-face care, including from general practitioners (87%), and had been prescribed psychotropic medication (79.2%). A notable finding was the high number who had recently experienced bereavement, mainly in the form of the end of a relationship (31.4%) (Table 2).

**Table 2:** Data from National Coronial Information System documents on study patients who died by suicide (*n* = 90)^a^

	*n*	%
Total cases linked by the Australian Institute for Health and Welfare	90	
Coroners’ reports available	81^a^	
Documented depressive illness (inc. that associated with borderline personality disorder)	66/81	81.5%
Psychotic disorder (schizophrenia, bipolar disorder, OCD)	18/81	22.2%
Documented substance use disorder (inc. alcoholism)	15/81	18.5%
Documented previous suicide attempt	42/69^b^	60.9%
Recent (last 6 months) hospital admission	36/69	52.2%
Recent separation or bereavement	22/70	31.4%
Face-to-face mental healthcare (inc. GP prescription of antidepressants)	67/77	87.0%
Current psychotropic prescription (inc. antipsychotics, mood stabilisers)	61/77	79.2%
Toxicology^c^		
Alcohol present	32/77	41.6%
Benzodiazepine present	24/77	31.2%
SNRI antidepressant present	20/77	26.0%
SSRI antidepressant present	18/77	23.4%
Tricyclic antidepressant	9/77	11.7%
Antipsychotics and mood stabilisers	14/77	18.2%
Illegal drugs (cannabis, methamphetamine, non-prescribed opioids)	16/77	20.8%

GP, general practitioner; inc., including; OCD, obsessive–compulsive disorder; SNRI, serotonin–noradrenaline reuptake inhibitor; SSRI, selective serotonin reuptake inhibitor.

a. No coroner/police report was found on 9 cases.

b. Limited psychiatric history in 12 coroner/police reports.

c. Toxicology available on only 77 cases.

### Toxicology reports in NCIS documents

There were toxicology reports for 77 of 81 cases in which NCIS documents could be found, of which four-fifths (79.2%) showed the presence of prescribed medications. A higher proportion had serotonin–noradrenaline reuptake inhibitor antidepressants (SNRIs) (26%) than selective serotonin reuptake inhibitors (SSRIs) (23.4%) or tricyclic antidepressants (TCAs) (11.7%), although in several cases the coroners’ reports confirmed that TCAs were taken as a lethal overdose, rather than because they were the patient's usual treatment. Alcohol was found to be present in 32 cases (41.6%) and illegal drugs in 16 (20.8%). However, another finding of concern was the high percentage (31.2%) of cases in which benzodiazepine medication was found in the toxicology report.

### Cluster analysis

**Fig. 2:**
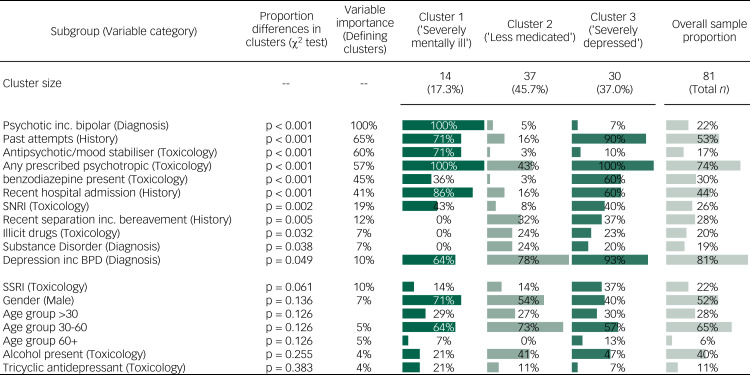
Cluster analysis of factors associated with subsequent suicide. BPD, bipolar disorder; SNRI, serotonin–noradrenaline reuptake inhibitor; BPD, bipolar disorder; inc., including; SSRI, selective serotonin reuptake inhibitor.

Variables from the 81 cases for which documents were available in the NCIS, including the results of the toxicology reports, were subjected to a two-step cluster analysis, which revealed three main clusters, loosely described as ‘severely mentally ill’, ‘severely depressed’ and ‘less medicated’ (Fig. 2) The severely mentally ill cluster included a diagnosis of schizophrenia, bipolar disorder or severe obsessive–compulsive disorder treated with antipsychotic medication; a high proportion in this cluster were taking psychotropic medication, including antipsychotics and mood stabilisers, and had a recent hospital admission and previous suicide attempt. The severely depressed cluster were also all taking medication, including a higher proportion on an SNRI antidepressant or benzodiazepine medication, and had an even higher rate of documented previous suicide attempt. The cluster termed ‘less medicated’ were known to have been depressed according to the coroners’ reports, but were less likely to have a documented previous suicide attempt, a psychiatric hospital admission or treatment with medication, including both antidepressants and benzodiazepines. Recent separation and bereavement was a prominent finding in both the severely depressed and the less medicated groups.

## Discussion

This review of the circumstances of former patients of a DMHS found that those who died by suicide were often severely unwell, with a high proportion known to have depression, severe mental illness, previous suicide attempt and recent hospital admission. They were also mostly in contact with face-to-face services, already receiving treatment for mental illness and, based on the toxicology reports, were at least partly adherent to treatment at the time of death.

Despite the large number of patients who registered with the clinic reporting severe symptoms of depression and suicidal thoughts and plans, only a small number died by suicide soon after contact with the service. The mean duration from contact to suicide was around 18 months, reflecting the chronic nature of their conditions. The NCIS reports indicated that almost one-third had experienced recent separation or bereavement, and toxicology reports indicated that many were affected by disinhibiting substances at the time of their death.

The overall suicide rate of 56.6/100 000 per year for the 2 years after last contact with MindSpot is around five times that for the wider community, but is comparable to the rate of subsequent suicide of 47.5/100 000 per year reported among primary care and out-patient mental health service patients by Simon and colleagues.^16^ In that study of 509 945 patients who completed a PHQ-9 after reporting mood symptoms, there were 484 suicide deaths in the 2 years after contact, despite the interventions of treating clinicians who were aware of the patients’ responses.^16^

### Psychiatric diagnosis and suicide

The finding that 81.5% of the patients who died by suicide in this study were known to have a depressive illness was consistent with the findings of a systematic review of 76 studies of psychological autopsies, in which around 90% of those who died by suicide were known to have a pre-existing mental disorder, mainly depressive illness.^27^ In addition to diagnosed depression, 18/81 (22%) of those who died by suicide in our study had a severe mental illness, based on the reported diagnosis, the medications prescribed and the history of recent hospital admission. Comorbid substance misuse was known to be present in 38% of deaths by suicide in the systematic review of psychological autopsies^27^ and in a similar proportion in a large Australian study that also used NCIS data;^28^ this is a little higher than the diagnoses of substance use disorder recorded in our study, although 41.6% of the present sample had alcohol present and 20.8% had illegal drugs present at the time of death. The results suggests that substance use, particularly the use of substances likely to exacerbate low mood, reduce inhibition and increase the tendency to act on impulse (such as alcohol and amphetamine), is a significant risk factor for suicide. The finding that nearly one-third of those for whom toxicology reports were available had benzodiazepine medication in their bloodstream was of particular concern, given the comparatively low rate of benzodiazepine use in a later MindSpot Clinic sample (2020), 2.1% of whom reported taking regular or as required benzodiazepine medication.^23^ A review of 17 studies found that benzodiazepine use increases the overall risk of attempting and completing suicide,^29^ possibly by increasing impulsivity and reducing inhibition, as well as contributing to the lethal effect of any overdose.

### Contact with mental health services and suicide

This cohort was atypical of the results of primary studies and meta-analyses of contact with mental health services at the time of suicide, with three-quarters known (based on the presence of prescribed medication in toxicology) to be in current contact with at least primary healthcare, more than half having been in a psychiatric hospital in the previous 6 months, some of whom were current in-patients or only recently discharged. Hence the former DMHS patients who died by suicide included a higher proportion of severely ill treatment-seeking individuals than population-based samples.

Furthermore, in nearly every case the former patients who died by suicide were either engaged with a face-to-face mental health service or saw a clinician in person between their engagement with MindSpot and death. The finding that 90 former patients from the first 4 years of operation had died by suicide was initially alarming. However, the study has found that those who died had the most severe psychiatric disorder and that there were intervening events, in particular bereavement and substance use, that could not have been predicted by the clinic safety protocols or prevented by the clinic interventions.

### Relevance of NCIS data to DMHS suicide risk assessment

The strongest association with subsequent suicide appeared to be the presence of severe mental illness, as only a small proportion (3%) of the overall clinic sample in 2020 reported taking antipsychotic and mood stabilising medication.^23^ The main modifiable risk factors were substance use, including the use of alcohol, and treatment with benzodiazepine medication. MindSpot does not routinely discuss medication use with patients, and instead recommends attending treating doctors for review. However, when the topic arises, MindSpot therapists generally discourage reliance on benzodiazepine medications, because they can interfere with learning-based treatments and the mastery of anxiety symptoms using cognitive and behavioural strategies. Similarly, alcohol use is only discussed with patients as it arises. Another prominent finding is the effect of separation on people who already have severe depression. The presence of severe mental illness, disinhibiting substances, bereavement and the intervention of face-to-face mental health services in the average of 18 months after last contact suggests that DMHS have limited capacity to intervene to prevent suicide. Moreover, the comparatively small number of suicides among the 285 patients who were urgently referred for face-to-face care suggests that where interventions did occur, they were effective in addressing the presence of suicidal behaviour or the reasons for disclosing suicidal thoughts.

### Limitations

The study has several limitations. The first is that some of the people registered with MindSpot during the study period did not give consent for their data to be analysed, and a small number did not provide enough identifiable information for data linkage. Moreover, no documents were found in the NCIS for 9 included patients, and for a further 12 there was no detailed psychiatric history, which may have altered the results and the findings of the cluster analysis, which is any case is descriptive rather than predictive. The roll out of My Health Record, a national digital medical record in Australia, may allow a more accurate view of both face-to-face care and adherence to treatment in patients who die by suicide. It should also be noted that the period between the last contact and time of death in the current study ranged from 1 day to more than 5 years, and the average duration from last contact to death may be shorter, although the average duration between contact and death was still about a year in the earlier study examining a defined 2-year period.^13^ The current study was unable to estimate how many suicides may have been prevented by effective treatment of depression in those who enrolled in a treatment course, although treatment patients had a lower risk of suicide in the first 2 years (OR = 0.38) and urgent referral by MindSpot clinicians, who are themselves all registered mental health clinicians with extensive training, supervision and are subject to clinic protocols, to face-to-face services after the disclosure of suicidal thoughts was also associated with a comparatively low incidence of subsequent suicide. The study only considered patients who enrolled with MindSpot up to the end of 2016, mainly because of the delay in finalising coroners’ findings. The sample was collected prior to the COVID-19 pandemic and related economic upheaval, although the rate of suicide in Australia fell in the first year of the pandemic and has only recently returned to pre-pandemic levels. Moreover, there have been only minor changes in systems for assessment and engagement and in the treatments offered by MindSpot in the years since the sampling period. Another important limitation is that the results reported here are based on a well-governed DMHS in which services are delivered by trained mental health professionals, and may not generalise to fully automated services or services provided by unqualified staff.

Despite these limitations, the study provides important information about the type of patients who died by suicide after contact with a national DMHS, and the circumstances of death, which are similar to those faced by other mental health services. The results support the continued provision of DMHS aligned with appropriate regulatory frameworks. These results also support greater integration of DMHS with the wider mental health sector to provide more coordinated support for those with severe and complex needs.

### Clinical implications

Although our study found very few suicides at the time of or soon after contact with the MindSpot DMHS, it identified a group of patients who are of particular concern, including the recently bereaved, especially separated men, and people who misuse disinhibiting substances that might increase the risk of suicide while affected. This indicates the need to address the enduring risk of suicide in this group, either by follow-up or referral for further (in some cases ongoing) care. The inherent limitations of suicide risk assessment,^30,31^ in particular in our ability to predict intervening events such as exacerbations of illness, bereavement and substance use, and the clear benefit to most individuals who engage in treatment or accept urgent referral, indicate that if protocols are followed, the likelihood of subsequent suicide among individuals seeking assessment and treatment from a well-governed DMHS appears to be similar to that for face-to-face services, especially when the severity and complexity of the conditions of many of the people who accessed MindSpot is taken into account.

## Data Availability

Deidentified data that support these findings are available from the corresponding author on reasonable request.
